# Immunization of Chickens with the Enterobactin Conjugate Vaccine Reduced *Campylobacter jejuni* Colonization in the Intestine

**DOI:** 10.3390/vaccines8040747

**Published:** 2020-12-09

**Authors:** Yifang Cui, Fangfang Guo, Jie Guo, Xiaoya Cao, Huiwen Wang, Bing Yang, Hongzhuan Zhou, Xia Su, Ximin Zeng, Jun Lin, Fuzhou Xu

**Affiliations:** 1 Beijing Key Laboratory for Prevention and Control of Infectious Diseases in Livestock and Poultry, Institute of Animal Husbandry and Veterinary Medicine, Beijing Academy of Agricultural and Forestry Sciences, Beijing 100097, China; cuiyifang@iasbaafs.net.cn (Y.C.); guofangfang@iasbaafs.net.cn (F.G.); guojie@iasbaafs.net.cn (J.G.); caoxiaoya@iasbaafs.net.cn (X.C.); yangbing@iasbaafs.net.cn (B.Y.); zhouhongzhuan@iasbaafs.net.cn (H.Z.); suxia@iasbaafs.net.cn (X.S.); 2 Department of Animal Science, The University of Tennessee, Knoxville, TN 37996, USA; hwang83@vols.utk.edu (H.W.); xzeng3@utk.edu (X.Z.)

**Keywords:** enterobactin, conjugate vaccine, *Campylobacter jejuni*, chicken, colonization

## Abstract

*Campylobacter jejuni* is the leading bacterial cause of human enteritis in developed countries. Chicken is the major animal reservoir of *C. jejuni* and a powerful infection model for human campylobacteriosis. No commercial vaccine against *C. jejuni* is available to date. The high affinity iron acquisition mediated through enterobactin (Ent), a small siderophore, plays a critical role in the colonization of *C. jejuni* in the intestine. Recently, an innovative Ent conjugate vaccine has been demonstrated to induce high-level of Ent-specific antibodies in rabbits; the Ent-specific antibodies displayed potent binding ability to Ent and inhibited Ent-dependent growth of *C. jejuni*. In this study, using specific-pathogen-free (SPF) chickens, we performed three trials to evaluate the immunogenicity of the Ent conjugate vaccine and its efficacy to control *C. jejuni* colonization in the intestine. The purified Ent was conjugated to the carrier keyhole limpet hemocyanin (KLH). Intramuscular immunization of chickens with the Ent–KLH conjugate for up to three times did not affect the body weight gain, the development of major immune organs and the gut microbiota. In the first two trials, immunizations of chickens with different regimens (two or three times of vaccination) consistently induced strong Ent-specific immune response when compared to control group. Consistent with the high-level of systemic anti-Ent IgG, *C. jejuni* colonization was significantly reduced by 3–4 log_10_ units in the cecum in two independent vaccination trials. The third trial demonstrated that single Ent–KLH vaccination is sufficient to elicit high level of systemic Ent-specific antibodies, which could persist for up to eight weeks in chickens. Taken together, the Ent–KLH conjugate vaccine could induce high-level of Ent-specific antibodies in chickens and confer host protection against *C. jejuni* colonization, which provides a novel strategy for *Campylobacter* control in poultry and humans.

## 1. Introduction

*Campylobacter jejuni,* one of the most important food-borne pathogens, is considered to be the most common bacterial cause of human gastroenteritis worldwide [1]. Additionally, the chronic sequelae of *C. jejuni* infection can lead to human autoimmune diseases such as Guillain–Barré syndrome and reactive arthritis [2]. Although these diseases are generally mild, they can be fatal among young children, elderly and immunosuppressed individuals [3]. Poultry is considered to be the major reservoir of *C. jejuni*. Consumption of raw or uncooked poultry is regarded as the major risk factor for human campylobacteriosis [4]. It was reported that poultry-related products cause about 60–80% of the global campylobacteriosis cases [3]. Colonization of chicken by *C. jejuni* can reach 10^6^–10^10^ CFU/g in the intestine [5]. To effectively control the rate of incidence of human infections, it is important to reduce the colonization level of *C. jejuni* in poultry [6,7]. However, due to complex interaction between *C. jejuni* and its host, limited progress has been made in the development of effective intervention strategies against *C. jejuni* in both poultry and humans [5,8].

Antibiotics are required for treating *Campylobacter* infections in humans [3]. Antimicrobial use in the poultry sector has usually followed the approach of using broad spectrum antibiotics (e.g., the antibiotics against Gram-negative bacteria to treat infections caused by avian pathogenic *Escherichia coli*) [9]. This has led to alarming increase of antimicrobial resistance (AMR) in both pathogenic and commensal bacteria present in the gut [10,11]. An example of this is the zoonotic pathogen *Campylobacter* spp. [12,13]. Thus, due to worldwide trend to limit antibiotic use in animal production, the non-antibiotic approaches, such as vaccination, are extremely important to prevent the colonization of *C. jejuni* in poultry, consequently reducing the rate of incidence of human infections [14,15]. Multiple vaccine development strategies including whole-cell killed vaccines, subunit vaccines and delivery vector-based vaccines have been investigated in poultry [16]. However, to date, no vaccine has been approved globally to prevent *C. jejuni* infections [3]. 

Iron acquisition plays an important role in *C. jejuni* pathogenesis [17]. In particular, the enterobactin (Ent)-mediated high-affinity iron uptake system is considered to be essential for in vivo survival and colonization of *C. jejuni*, which has been demonstrated in chicken, an ideal infection model to study the colonization and immunogenicity of *C. jejuni* in the host [18,19,20]. Notably, inactivation of Fe-Ent receptors CfrA or CfrB significantly diminished and even abolished *C. jejuni* colonization in the intestine [18,19]. However, the vaccination strategies targeting the Fe-Ent receptor failed to confer protection against *C. jejuni* colonization in the host using chicken model [21,22]. Recently, an innovative Ent conjugate vaccine was developed; such vaccine could induce production of Ent-specific antibodies that showed inhibitory effect on *C. jejuni* growth in vitro [23]. Specifically, the Ent conjugate vaccine could trigger high level of Ent-specific antibodies in rabbits, which displayed similar bacteriostatic feature as lipocalins, the host innate immune effectors with potent Ent-binding ability [23]. Moreover, Wang et al. [23,24] demonstrated the Ent-specific antibodies significantly inhibited Ent-dependent growth of *Campylobacter* spp., *E. coli* and *Salmonella enterica*. However, to date, no information exists concerning protective efficacy of this novel Ent conjugate vaccine for *Campylobacter* control using appropriate animal model systems.

Here, we used chicken, a powerful infection model of *C. jejuni*, to examine the immune response and protective efficacy of the Ent conjugate vaccine against *C. jejuni* colonization. Specifically, Ent as a hapten was conjugated with keyhole limpet hemocyanin (KLH), a carrier protein, to prepare the Ent–KLH conjugate vaccine. Using specific-pathogen-free (SPF) chickens, we performed three trials to evaluate the immunogenicity of the Ent–KLH vaccine and its efficacy to control *C. jejuni* colonization in the intestine. The findings from the challenge trials with different vaccination regimens provided compelling evidence that the Ent–KLH conjugate vaccine could significantly reduce *C. jejuni* colonization in the chicken intestine, consistent with the drastically induced high level of Ent-specific antibodies in the chickens upon vaccination. 

## 2. Materials and Methods 

### 2.1. Ethics Statement

All chicken experiments were conducted in accordance with the guidelines approved by the Institutional Animal Care and Use Committee at Institute of Animal Husbandry and Veterinary Medicine, Beijing Academy of Agriculture and Forestry Sciences.

### 2.2. Bacterial Strain and Culture Conditions

*Campylobacter jejuni* NCTC 11168 was routinely grown in Mueller–Hinton (MH) broth (Difco, Sparks, MD, USA) or on agar at 42 °C in a microaerophilic incubator (5% O_2_, 10% CO_2_, 85% N_2_).

### 2.3. Enterobactin Purification and Conjugation

Enterobactin purification and conjugation procedures were performed as described previously [23]. Briefly, Ent was purified from the supernatant of an Ent transport mutant *E.scherichia coli* AN102. The purified Ent was dissolved in dimethylformamide (DMF) and coupled with KLH and bovine serum albumin (BSA) (Thermo Fisher Scientific, Waltham, MA, USA), respectively. Each batch of Ent–KLH and Ent–BSA conjugates was subjected to SDS-PAGE analysis to validate conjugation as described previously [23]. Finally, the purified Ent–KLH and Ent–BSA conjugates were adjusted to a concentration of 1 mg/mL in sodium phosphate buffer (PBS, 0.01 M, pH 7.2).

### 2.4. Enterobactin-Keyhole Limpet Hemocyanin (Ent–KLH) Conjugate Vaccine Preparation

The purified Ent–KLH conjugate (1 mg/mL) was emulsified with MONTANIDE™ RANGE (Seppic, Paris, France) in a volume ratio of 3:7 following the manufacturer’s instructions to prepare a water-in-oil emulsion for immunization in chicken.

### 2.5. Vaccination Procedures

All immunization experiments were performed using one-week-old SPF White Leghorn chickens (Beijing Boehringer Ingelheim Vital Biotechnology Co, Ltd., Beijing, China). All the chickens were determined to be negative for *Campylobacter* by culturing cloacal swabs prior to use, as described previously [18]. For each trial, the chickens were randomly divided into two groups in equal number. Each group was individually housed and fed ad libitum. Each chicken in the treatment group was immunized with 100 μg of Ent–KLH conjugate vaccine via intramuscular injection (inner thigh) while the chickens in the control group were immunized with PBS solution (0.01 M, pH 7.2) emulsified with the same adjuvant.

**Figure 1:**
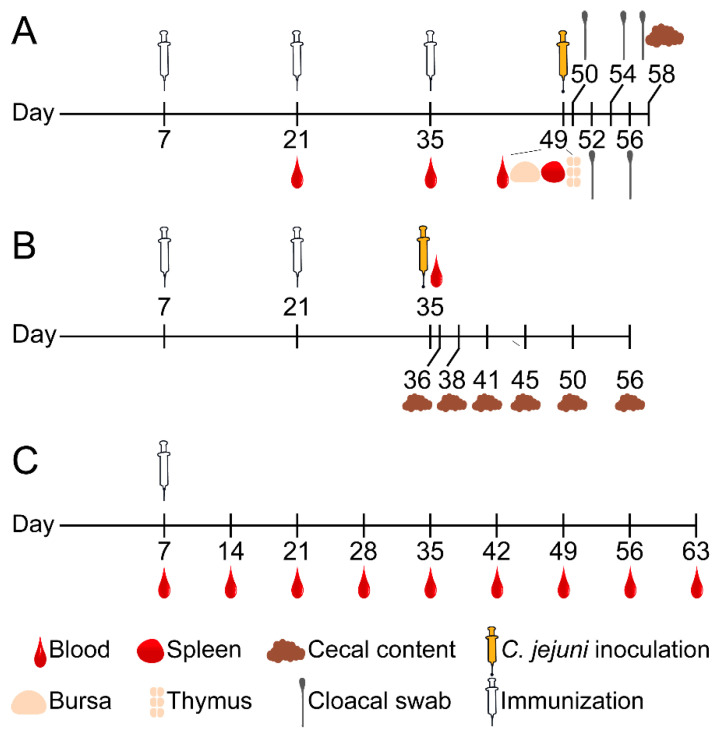
Diagram of three chicken immunization trials. For each trial (20–30 SPF chickens per group), each chicken in the treatment group was immunized with 100 μg of Ent–KLH conjugate emulsified with MONTANIDE™ RANGE adjuvant (Seppic, Paris) via intramuscular injection (inner thigh) while the chicken in control group was immunized with PBS solution (0.01 M, pH 7.2) emulsified with the same adjuvant. (**A**) Chickens (25 birds per group) received three immunizations at age of 7, 21 and 35 days, respectively. Blood samples were collected 14 days after each of immunizations. Each chicken was weighed weekly. Nine chickens of each group were euthanized at 49 days old; major immune organs (bursa, spleen and thymus) and cecal contents were collected from each chicken as well. The remaining 16 chickens of each group were orally challenged with *C. jejuni* (10^4^ CFU per chicken) two weeks after the last immunization. Cloacal swabs were collected from each chicken every 1–2 days post inoculation and cecal contents were also collected upon termination for CFU enumeration. (**B**) Chickens (30 bird per group) received two immunizations at age of 7 and 21 days, respectively. Blood samples were collected 14 days after the second immunization. All chickens were orally challenged with *C. jejuni* (10^4^ CFU per chicken) two weeks after the second immunization. Five chickens in each group were euthanized at indicated age post challenge and cecal contents were collected from each chicken for CFU enumeration. (**C**) Chickens (20 birds per group) only received single immunization at age of one week old. Blood samples were collected immediately prior to immunization and weekly after the immunization until the chickens were euthanized at age of 63 days.

In Trial A, 50 SPF chickens (25 chickens per group) were used to evaluate the effects of immunization of chickens with the Ent–KLH vaccine for three times on growth performance, Ent-specific immune response and protection against *C. jejuni* colonization (Figure 1A). Chickens received three immunizations at age of 7, 21 and 35 days, respectively. Blood samples were collected 14 days after each immunization and the sera were separated for detection of Ent-specific antibody. Each chicken was weighted weekly. Nine chickens of each group were euthanized at 49 days old; major immune organs (bursa, spleen and thymus) and cecal contents were collected from each chicken as well. The development of each immune organ was determined according to the percentage of each immune organs (bursa, spleen or thymus) weight to its body weight at 49 days old. Cecal content from each euthanized chicken was immediately frozen in liquid nitrogen for microbiota analysis described below. The remaining 16 chickens in each group were orally challenged with *C. jejuni* (10^4^ CFU per chicken) at age of 49 days old. Cloacal swabs were collected from each chicken on Days 1, 3, 5, 7 and 9 post inoculation. On Day 9 post inoculation, all chickens were euthanized and cecal contents were collected. Both cloacal swabs and cecal samples were subjected to 10-fold serial dilutions and plated on MH agar plates containing *Campylobacter*-specific selective supplements (SR117E, Oxoid, Bashingstoke, Hampshire, England) for enumeration of *C. jejuni* as described previously [18].

In Trial B, 60 SPF chickens (30 chickens per group) were used to determine immune response and protective efficacy of Ent–KLH conjugate vaccine for reducing *C. jejuni* colonization in chicken (Figure 1B). In this trial, chickens only received two immunizations at age of 7 and 21 days, respectively, followed by *C. jejuni* challenge. Blood samples were collected from all chickens 14 days after the second immunization and immediately prior to challenge for assessing Ent-specific antibody. All the chickens were orally challenged with *C. jejuni* (10^4^ CFU per chicken) at age of 35 days old. Subsequently, five chickens in each group were euthanized on Days 1, 3, 6, 10, 15 and 21 post inoculation, respectively, for collecting cecal contents. The cecal contents were used for CFU enumeration of *C. jejuni* as described above.

In Trial C, 40 SPF chickens (20 chickens per group) were used to assess the persistence of Ent-specific antibodies upon single vaccination of chickens with the Ent–KLH conjugate vaccine (Figure 1C). Chickens only received single immunization at age of one week old. Blood samples were collected immediately prior to immunization and weekly after the immunization until the chickens were euthanized at age of 63 days. All the collected sera were subjected to ELISA analysis for detection of Ent-specific antibody.

### 2.6. Analysis of Gut Microbiota

Total DNA was extracted from each cecal sample using a Monarch^®^ Genomic DNA Purification Kit (NEB, Ipswich, MA, USA) in accordance with the manufacturer’s protocols. The DNA extracts were subjected to microbial community analysis using multiplex tag-encoded 16S rRNA gene (rDNA) amplicon sequencing, which was performed by Majorbio Bio-Pharm Technology Co., Ltd. (Shanghai, China) using Illumina Miseq platform. Raw data were demultiplexed and quality filtered by Trimmomatic. The 16S rDNA amplicon sequences were analyzed in terms of taxonomic classification as well as grouped in operational taxonomic units (OTUs) at different sequence similarity levels.

### 2.7. Enzyme-Linked Immunosorbent Assay (ELISA)

An indirect ELISA assay was used to examine Ent-specific antibody in serum samples as described previously with some modifications [23]. Briefly, given the lack of cross reactivity between KLH and BSA, the Ent–BSA was used as coating antigen for the detection of specific anti-Ent IgG level in the chickens immunized with Ent–KLH vaccine. The microplates were coated with 100 μL of Ent–BSA solution (2 ng/μL) in coating buffer (bicarbonate/carbonate coating buffer, pH 9.6) overnight at 4 °C. Each of the collected serum samples was diluted by 200-fold using blocking buffer (PBS containing 5% skim milk) and 100 μL of the diluted sample were added to each well. Each serum sample was performed in triplicate. After 1 h of incubation at 37 °C, the plates were washed three times with washing buffer (PBS containing 0.05% Tween 20). The HRP-labeled rabbit anti-chicken IgG (Millipore Sigma, St. Louis, MO, USA) was diluted 1:10,000 in blocking buffer, and 100 μL were added to each well as the secondary antibody. Following washing the plates three times with washing buffer, 100 μL of TMB (3, 3′, 5, 5′-tetramethylbenzidine) substrate (Beyotime Biotechnology, Shanghai, China) were added in each well and incubated at 37 °C for 30 min for color development. The reaction was stopped using 1 M H_2_SO_4_ solution (50 μL per well) and the absorbance was measured at OD_450_ using an ELISA reader (Synergy H1, BioTek, Winooski, VT, USA).

### 2.8. Statistical Analysis

Statistical analyses were conducted using an unpaired t test with two-tailed distribution. Data are presented as means ± the standard deviations. Results were considered significant if the *p* value was less than or equal to 0.05.

## 3. Results

### 3.1. Multiple Vaccinations of Chickens with the Ent–KLH Conjugate Vaccine Did Not Cause Phenotypic Changes in Physiology (Trial A)

**Figure 2:**
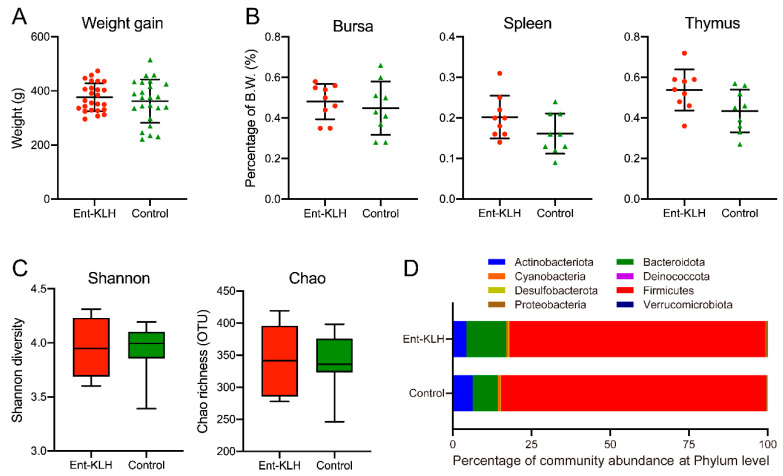
Effects of vaccination of chickens with the Ent–KLH conjugate on growth performance, immune organ development, and gut microbiota. (**A**) Chicken body weight gain. Each symbol represents the body weight gain of each individual chicken between ages of 7 and 49 days. (**B**) The weight percentage of each immune organ (bursa, spleen or thymus) to its body weight at 49 days old. (**C**) The Shannon diversity and the Chao richness (OTU) based on 16S rDNA amplicon sequences of the cecal samples. (**D**) Relative abundance of the eight dominant phyla in the cecum between Ent–KLH and PBS-control groups.

One goal of Trial A was to determine if up to three vaccinations of chickens with the Ent–KLH conjugate influenced chicken physiology with respect to body weight gain, development of major immune organs (bursa, spleen and thymus) and composition of gut microbiota when compared to control group. As shown in Figure 2A, no significant difference was observed in body weight gain for chickens between the two groups (*p* = 0.65). The development of major immune organs, including bursa (*p* = 0.61), spleen (*p* = 0.14) and thymus (*p* = 0.13) also showed no significant difference between the two groups (Figure 2B). Microbiota analysis of the cecal contents from the chickens in two groups did not reveal significant differences regarding Shannon (*p* = 0.11) and Chao (*p* = 0.93) indices, which represent the bacterial diversity and abundance, respectively (Figure 2C). The diverse bacterial phyla were identified in the cecal contents from both groups. The dominant phyla identified in Ent–KLH and control groups were Firmicutes (81.17% vs. 84.15%), Bacteroidetes (12.71% vs. 7.90%), Actinobacteria (4.26% vs. 6.33%), Cyanobacteria (0.69% vs. 0.84%), Proteobacteria (0.90% vs. 0.46%), Desulfobacterota (0.27% vs. 0.28%), Verrucomicrobiota (0.0002% vs. 0.0003%) and Deinococcota (0.00001% vs. 0.00001%). No significant difference was observed among the eight phyla between the two groups (*p* > 0.99) (Figure 2D).

### 3.2. The Ent–KLH Conjugate Vaccine Significantly Elicited Ent-Specific Antibody Response (Trial A)

**Figure 3:**
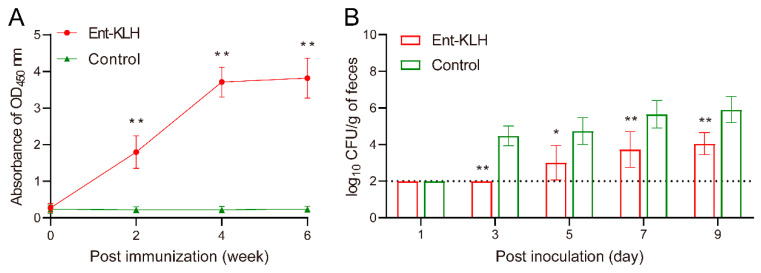
Enterobactin-specific immune response and protective efficacy of the Ent–KLH conjugate vaccine to mitigate *C. jejuni* colonization in the chicken intestine (Trial A with a total of three immunizations). (**A**) The Ent-specific IgG in the chickens immunized with Ent–KLH or PBS. Each point represents the average of OD_450_ absorbance from 25 individual serum samples (200-fold dilution) with standard deviation indicated by error bar. (**B**) Comparison of colonization of *C. jejuni* NCTC 11168 in the chickens immunized with Ent–KLH or PBS. Each bar represents mean CFU/g feces ± the standard deviation in each group. The dotted line indicates detection limit. * *p* < 0.05, ** *p* < 0.01.

To accurately determine the level of Ent-specific antibodies rather than those directed against whole conjugate vaccine in serum samples, the Ent–BSA conjugate was used as the coating antigen in the ELISA assay. Clearly, in Trial A, the chickens receiving three vaccinations displayed significantly (*p* < 0.01) higher level of anti-Ent antibodies than that the control group as early as two weeks after the first immunization (Figure 3A). The mean OD_450_ values of the serum samples from the Ent–KLH immunized chickens were 1.80, 3.71 and 3.80 at two weeks following the first, second and third immunization, respectively, while those from control group displayed significantly (*p* < 0.01) lower mean OD_450_ values (0.20–0.24). The Ent-specific antibody level at four and six weeks post immunization in treatment group did not show significant difference (*p* = 0.98) (Figure 3A).

### 3.3. Multiple Vaccinations of Chickens with the Ent–KLH Conjugate Reduced Colonization of C. jejuni in Chicken Intestine (Trial A)

In Trial A, due to removal of nine chickens at age of 49 days old for sample collections (Figure 1A) and corresponding analyses (Figure 2), there were 16 birds left for *C. jejuni* challenge following three immunizations. To better monitor colonization dynamics and pattern of *C. jejuni* in the intestine, cloacal samples were collected from each bird for CFU enumeration at five different time points post inoculation. As shown in Figure 3B, the *C. jejuni* level in the feces from the chickens receiving Ent–KLH vaccinations, based on the assumption of 100 mg of feces per swab, were significantly lower than those from control chickens from three days post inoculation; the difference ranged from 1.75 to 2.73 log_10_ units. Notably, upon termination of the trial (nine days post inoculation), cecal contents were also collected from each bird in both groups for accurate enumeration of *C. jejuni* in the intestine, which showed that vaccination of chickens with Ent–KLH led to significant *C. jejuni* reduction (>4 log_10_ units) in cecum (4.61 log_10_ units) when compared to control group (8.65 log_10_ units) at nine days post *C. jejuni* challenge.

### 3.4. Different Vaccination Regimen Still Significantly Induced High Level of Ent-Specific Antibodies and Reduced Colonization of C. jejuni in the Chicken Intestine (Trial B)

To confirm the efficacy of the Ent–KLH vaccine observed in Trial A, a complementary chicken experiment (Trial B) was performed with following three modifications: (1) the chickens in treatment group only received two immunizations; (2) more chickens in each group (30 birds per group) were subjected to *C. jejuni* challenge so that five birds were euthanized at each of the six time points post inoculation for accurate assessment of *C. jejuni* level in the cecum, which overcame the limitation of semi-quantitative nature of using cloacal swabs in Trial A (Figure 3B); and (3) the time period for intestinal sample collection following *C. jejuni* challenge was greatly extended (up to 21 days post inoculation) when compared to Trial A (up to nine days post inoculation).

**Figure 4:**
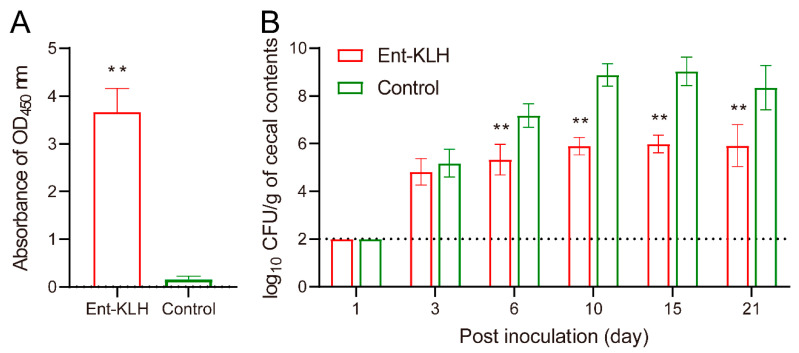
Enterobactin-specific immune response and protective efficacy of the Ent–KLH conjugate vaccine to mitigate *C. jejuni* colonization in chickens (Trial B with two immunizations). (**A**) The Ent-specific IgG in the chickens immunized with Ent–KLH or PBS. Each bar represents the average of OD_450_ absorbance from 30 individual serum samples (200-fold dilution) with standard deviation indicated by error bar. (**B**) Comparison of colonization of *C. jejuni* NCTC 11168 in the chickens immunized with Ent–KLH or PBS. Each bar represents mean CFU/g cecal contents ± the standard deviation in each group. The dotted line indicates detection limit. ** *p* < 0.01.

Despite different vaccination regimen used in Trial B (two immunizations), the mean OD_450_ value of the serum samples from the Ent–KLH immunized chickens (3.66) at two weeks following the second immunization was significantly higher (*p* < 0.01) than that from control group (0.16) (Figure 4A), indicating the reduced frequency of Ent–KLH vaccination still induced desired level of Ent-specific antibodies in the system.

Quantitative measurement of *C. jejuni* in cecal contents at different time points post inoculation (Figure 4B) confirmed the protective efficacy of the Ent–KLH vaccine observed in Trial A (Figure 3B): the *C. jejuni* level in the chickens immunized with the Ent–KLH was significantly (*p* < 0.01) lower than that in control group (up to 3.20 log_10_ units) from six days post inoculation (Figure 4B). Notably, at 21 days post inoculation, the *C. jejuni* colonization level in the cecum of the chickens receiving the conjugate vaccine was still more than 2 log_10_ units lower than that in control group (Figure 4B).

### 3.5. Persistence of the Ent-Specific Antibodies Upon Single Vaccination of Chickens with the Ent–KLH Conjugate Vaccine (Trial C)

**Figure 5:**
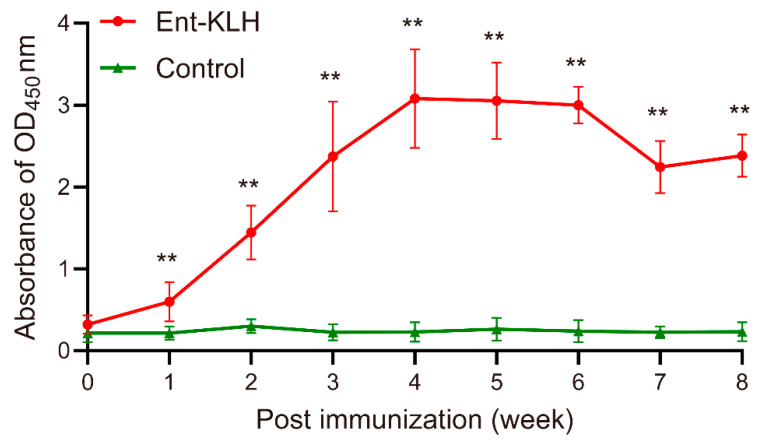
Persistence of Ent-specific IgG upon single vaccination of chickens with the Ent–KLH conjugate vaccine (Trial C). Each point represents the average of OD_450_ absorbance from 20 individual serum samples (200-fold dilution) with standard deviation indicated by error bar. ** *p* < 0.01.

The findings from Trial B strongly suggest that the chickens receiving reduced times of vaccination could still produce high level of Ent-specific antibodies, which could significantly mitigate *C. jejuni* colonization for a long time. To further optimize vaccination regimen for induction of desired immune response, in Trial C, chickens were vaccinated with the Ent–KLH conjugate one time at seven days old and the level and persistence of the Ent-specific antibodies in serum were monitored weekly for eight weeks post immunization. The mean OD_450_ values of sera from Ent–KLH group were significantly higher than those in the control group as early as one week post immunization (*p* < 0.01) (Figure 5). The Ent-specific antibody level in Ent–KLH group reached a peak at four weeks post immunization (mean OD_450_ = 3.08), sustained for at least two weeks and then dropped to mean OD_450_ of 2.24 and 2.38 at seven and eight weeks post immunization, respectively (Figure 5).

## 4. Discussion

Iron is an essential micronutrient for all organisms [25]. During the “tug-of-war” for iron between bacteria and host, the competition for iron is so intense that microbial iron acquisition systems are major determinants of virulence [25,26]. Siderophore-mediated iron uptake system plays an important role in modulation of bacteria colonization and growth in the host [27]. Currently, three strategies were developed to inhibit siderophore biosynthesis or utilization: (1) screening inhibitors of siderophore biosynthetic enzymes as novel antimicrobials [28]; (2) design of siderophore–antimicrobial conjugates against antimicrobial resistance [29,30]; and (3) development of siderophore-based conjugate vaccines [23,31,32].

Siderophore-based immunization strategy is promising to inhibit growth of different Gram-negative pathogens, such as *E. coli* and *Salmonella* infections in mice [31,32]. In the study by Mike et al. [32], the siderophore conjugate vaccine failed to elicit any detectable siderophore-specific antibodies in mice. Relevant to this work, Sassone-Corsi et al. [31] reported an Ent conjugate vaccine that was synthesized using a lengthy and complicated procedure; this Ent conjugate elicited weak mucosal response (i.e., <4-fold increase in Ent-specific antibodies) and no systemic response (IgG) in the immunized mice. In our recent study [23], we developed a new Ent conjugate vaccine using a simple and efficient protocol. This new vaccine could trigger strong systemic immune responses, resulting in up to 4,096-fold increase in the titer of Ent-specific IgG in serum. In addition, the Ent-specific antibodies induced by this novel Ent conjugate vaccine in rabbit could inhibit Ent-dependent growth of diverse *Campylobacter*, *E. coli* and *S. enterica* in vitro [23,24]. Therefore, the new Ent conjugate vaccine is promising for controlling *Campylobacter* and other Gram-negative pathogens in poultry. This study, for the first time, demonstrated that the Ent–KLH conjugate vaccine could elicit high level of systemic Ent-specific antibodies in chickens and significantly reduce *C. jejuni* colonization in the intestine, which was confirmed in two independent chicken trials (Figure 3 and Figure 4). In addition, immunization of the Ent–KLH conjugate vaccine had no effects on the body weight gain and the development of major immune organs (Figure 2A,B). These data indicate that the Ent–KLH conjugate vaccine has high potential for application in poultry industry to control infections caused by *Campylobacter* and even other Gram-negative pathogens. 

As Ent is produced by most members of Enterobacteriaceae [27], the potential effects of an Ent conjugate vaccine on the composition and abundance of the gut microbiota should be investigated in animal experiments. Our results show that the diversity and abundance of the chicken gut microbiota were not affected by three times of vaccination of chickens with the Ent–KLH conjugate (Figure 2C,D). Previous study also showed that immunization of mice with the Ent-CTB conjugate vaccine did not significantly influence the composition of microbiota in mice without intestinal inflammation [31]. Moreover, reduction of *Salmonella* colonization was accompanied by significant expansion of *Lactobacillus* spp. in the inflamed gut of mice immunized with CTB-Ent conjugate vaccine, which provided additional benefits to the host by indirectly promoting the expansion of beneficial microbes in the inflamed gut [31]. Extensive microbiome studies have consistently shown that poor gut health is associated to the expansion of dysbiotic Proteobacteria [33], the most important bacterial population producing catecholate siderophores. Therefore, the Ent conjugate vaccine examined in this study may be beneficial for enhancing host gut health by controlling overpopulation of Proteobacteria species and by expanding beneficial microbes.

Stimulation of specific and sustained immune response is an important criteria for evaluating the protective efficacy of the vaccine [34]. As discussed above, the new Ent–KLH conjugate vaccine reported by Wang et al. [23] has shown significant advantage over other siderophore conjugate vaccines with respect to induction of high level of siderophore-specific antibodies [31,32]. The findings from three vaccination trials in this study further provided compelling evidence that intramuscular immunization of chickens with the Ent–KLH conjugate vaccine, regardless of vaccination regimens, significantly elicited Ent-specific antibodies (Figure 3A, Figure 4A and Figure 5). Another new and exciting finding in this study is that the high level of Ent-specific antibodies induced by single Ent–KLH vaccination could persist for up to eight weeks post immunization (Figure 5). This feature, which clearly makes this vaccine more economically feasible for application in poultry industry, may provide long-term protection of chickens against *Campylobacter* infections. This speculation needs to be examined in the future.

In addition to using the Ent–KLH conjugate vaccine to induce active immunity, passive immunization of food-producing animals and even humans with Ent-specific hyperimmune egg yolk antibodies is another promising approach to prevent and control *Campylobacter*. Laying hen has been recognized as an efficient “bioreactor” to produce a large quantity of specific egg yolk antibodies; therefore, passive immunization with specific egg yolk antibodies is regarded as a potential alternative to antibiotics for control of various infectious diseases [35]. For cost-efficient production of large quantities of specific egg yolk antibodies, it is essential to elicit high and steady immune response in laying hens. The findings from multiple vaccination trials in this study, particularly Trial C (Figure 5), demonstrated that single immunization of chickens with the Ent–KLH vaccine could trigger high-level of systemic Ent-specific antibodies sustaining for up to eight weeks. Thus, the finding from this study may provide a simple immunization procedure for efficient production of Ent-specific antibody in laying hens.

Consistent with previous in vitro finding that Ent-specific antibodies significantly inhibited the Ent-dependent growth of *Campylobacter* [23], the immunization-challenge trials in this study demonstrated that the Ent–KLH conjugate vaccine can reduce *C. jejuni* loads by up to 10,000-fold in chicken cecum, which exhibits a better protective efficacy than most reported vaccination strategies for *C. jejuni* control in chickens [8,36]. In addition to the novelty of the Ent–KLH conjugate vaccine and its potent protection against *C. jejuni* colonization in the chicken intestine, this study has some advantages in terms of experimental design when compared to previous chicken vaccination studies [8,36]. For example, the protective efficacy of the Ent–KLH was consistently observed in independent trials using different vaccination regimens. In addition, we showed long-term persistence of induced Ent-specific antibodies upon single immunization in this study, which is critically important for both active immunization and passive immunization approaches using the Ent–KLH conjugate vaccine in poultry. It is important to mention that high-quality SPF chickens were used for all trials in this study, which generates consistent and insightful findings for the immunogenicity and protective efficacy of the Ent–KLH vaccine in chickens. In the future, trials using commercial broilers are highly warranted to confirm the efficacy of the Ent-based immune intervention for *Campylobacter* control in poultry.

## 5. Conclusions

Chickens are a major source of human campylobacteriosis; however, there is no vaccine available for *Campylobacter* control in poultry. In this study, immunization of chickens with the innovative Ent–KLH conjugate vaccine using two different vaccination regimens consistently reduced *C. jejuni* colonization by 3–4 log_10_ units in the cecum. The Ent–KLH vaccine could significantly induce Ent-specific immune response in chickens but did not affect growth performance, development of immune organs and gut microbiota. Moreover, the high level of systemic Ent-specific antibodies due to single immunization could persist for eight weeks in chickens. In conclusion, the results of this study strongly support that the Ent–KLH conjugate vaccine is promising for *Campylobacter* control in poultry, consequently protecting food safety and public health.
